# Effects of a Combined Zumba and Video Game–Based Cognitive Intervention on Cognitive Function Among Older Adults in Klang Valley, Malaysia: Semirandomized Trial

**DOI:** 10.2196/88479

**Published:** 2026-07-03

**Authors:** Yook Chin Chia, Sharifah Nadhira Syed Annuar, Jactty Chew, Yunli Lee, Wei-Gene Lim, Wei Zern Yip, Kristin-Ann Leong Zhe Mun, Michael Jenkins

**Affiliations:** 1 Department of Clinical Medicine and Surgery, Jeffrey Cheah Sunway Medical School, Faculty of Medical and Life Sciences, Sunway University, No. 5, Jalan Universiti, Bandar Sunway, 47500 Selangor Darul Ehsan, Malaysia, Petaling Jaya, Selangor, 47500, Malaysia, 60 374918622 ext 7410; 2 Department of Primary Care Medicine, Faculty of Medicine, University of Malaya, Kuala Lumpur, Kuala Lumpur, Malaysia; 3 Ageing, Health and Well-Being Research Centre, Jeffrey Cheah Sunway Medical School, Faculty of Medical and Life Sciences, Sunway University, Petaling Jaya, Selangor, Malaysia; 4 Department of Biomedical Sciences, Jeffrey Cheah Sunway Medical School, Faculty of Medical and Life Sciences, Sunway University, Petaling Jaya, Selangor, Malaysia; 5 Research Centre for Human-Machine Collaboration (HUMAC), Faculty of Engineering and Technology, Sunway University, Petaling Jaya, Selangor, Malaysia; 6 School of Psychology, Faculty of Medical and Life Sciences, Sunway University, Petaling Jaya, Selangor, Malaysia; 7 School of Psychology, Faculty of Science and Engineering, University of Nottingham Malaysia, Semenyih, Selangor, Malaysia

**Keywords:** cognitive aging, physical activity, cognitive stimulation, combined intervention, Montreal Cognitive Assessment, MoCA, older adults

## Abstract

**Background:**

Age-related cognitive decline can threaten independence in older adults, creating an urgent need for effective and practical preventive strategies. Nonpharmacological approaches such as physical activity, cognitive stimulation, and combined programs show promise, but their comparative effectiveness and the specific cognitive domains they influence are not yet clearly established.

**Objective:**

This study evaluated the effects of 3 nonpharmacological interventions. These consisted of a physical activity program using Zumba, a video game–based cognitive stimulation program, and a combined physical and cognitive program. The aim was to determine their effects on global cognition and specific cognitive domains in community-dwelling older adults in the Klang Valley of Malaysia. A secondary objective was to examine the influence of adherence on cognitive outcomes.

**Methods:**

A total of 96 participants (median age 69 y, IQR 65-72 y; n=63, 65.6% female) were assigned to 4 groups: physical (n=12), cognitive (n=23), combined (n=43), and control (n=18). Global cognitive performance was measured using the Montreal Cognitive Assessment at baseline and postintervention. Mixed ANOVA examined time and time × group effects. Additional nonparametric analyses (Kruskal-Wallis, Mann-Whitney *U*, Wilcoxon signed rank tests) were conducted under intention-to-treat and per-protocol (PP) frameworks. Adherence was defined as attending 50% or more of scheduled sessions, with PP analyses reclassifying participants accordingly.

**Results:**

Global cognition improved significantly over time across all groups (time main effect: *F*_1,92_=7.03, *P*=.008; time × group interaction: *F*_3,92_=0.57, *P*=.64). PP analyses showed moderate gains for participants attending 50% or more of cognitive sessions (*r*=0.46) and larger gains for those adhering to the combined intervention (*r*=0.66), with median Montreal Cognitive Assessment scores increasing by approximately 2 points in both adherent groups. Subdomain analyses indicated improvements in language (all groups), visuospatial function (combined group), and orientation (cognitive group).

**Conclusions:**

All intervention types were associated with improvements in cognitive performance over time, with comparable gains across groups. Participants who achieved at least moderate adherence (≥50%) to the cognitive or combined interventions demonstrated larger and more consistent within-group effect sizes, highlighting the importance of sustained engagement. These findings support the feasibility and potential cognitive benefits of structured, multimodal nonpharmacological interventions for community-dwelling older adults.

## Introduction

Malaysia is rapidly becoming an aging society. The proportion of the population aged 60 years and older is projected to increase from 11.6% in 2024 to 17.3% by 2040, surpassing the 15% threshold commonly used to define an aging population [1]. This demographic transition reflects a global trend, as many high- and middle-income countries face similar shifts toward older age structures [2]. Based on these projections, Malaysia will officially be classified as an aging society by 2040.

Globally, the prevalence of mild cognitive impairment (MCI) has been estimated at 19.7% among individuals aged 50 years and older [3]. In Malaysia, previous studies have reported even higher estimates, with approximately 21.1% of older adults affected [4,5]. More recently, a study conducted in Kuantan, Malaysia, found the prevalence of MCI among adults aged 60 years and older to be 18.7% [6]. Despite these substantial prevalence rates, most individuals remain undiagnosed until later stages of cognitive decline, with only 8% of those in the early stage of MCI receiving a formal diagnosis [7]. These findings have important implications for independence, health care use, and caregiver burden, contributing to the rising societal impact of dementia [8].

Lifestyle-based interventions are increasingly recognized as promising strategies for delaying cognitive decline, particularly given the limited effectiveness of pharmacological treatments [9]. Aerobic exercise has been associated with improvements in brain structure and function through mechanisms such as enhanced cerebral blood flow and improved regulatory effects on the brain. Sustained aerobic exercise increases heart rate, boosts cerebral perfusion, and promotes glucose metabolism in the brain [10]. Among the various modalities, Zumba represents a culturally engaging form of exercise that combines rhythm, coordination, and social interaction. Participation in Zumba classes has been shown to improve quality of life and visuospatial memory among older adults [11,12]. Zumba has been proposed as a feasible public health strategy due to its enjoyable and socially engaging nature [13], and its increasing popularity within Malaysian community settings further underscores its potential for broader implementation.

Parallel to this, technology-driven cognitive training programs, including video game–based interventions, have emerged as scalable and accessible tools for older adults. Evidence from computerized and video game–based cognitive training indicates that overall effects on cognitive performance in healthy older adults are positive but small. Reported benefits vary substantially across cognitive outcomes and are strongly influenced by intervention design characteristics, including delivery format and training intensity [14]. Improvements in specific domains are inconsistent, and transfer to global cognition remains variable, underscoring the importance of task design and intervention structure. With increasing digital literacy among urban-dwelling Malaysian older adults, including those in the Klang Valley, such approaches are becoming increasingly feasible.

Emerging research suggests that combining physical and cognitive interventions may have synergistic effects, as aerobic exercise provides a neurophysiological foundation for plasticity, while cognitive training directly stimulates neural networks [15]. However, studies directly comparing these approaches, particularly in Asian populations, remain limited.

The present semirandomized controlled trial was designed to address these gaps in the Malaysian context. Older adults living in the Klang Valley were allocated into physical activity (Zumba), cognitive stimulation (video game), combined intervention, or control groups within community settings. This study evaluated the effects of the interventions on global cognitive function using the Montreal Cognitive Assessment (MoCA). We also explored changes across cognitive subdomains, examined whether attendance influenced outcomes, and identified participants who demonstrated meaningful improvements, defined as achieving the minimal clinically important difference of a ≥2-point increase in MoCA scores [16]. Thus, this study aimed to evaluate the effects of Zumba, video game–based cognitive stimulation, and combined interventions on global cognitive function among older adults in Klang Valley, Malaysia, and to examine the influence of adherence on cognitive outcomes, with the hypothesis that the combined intervention would produce the greatest improvements.

## Methods

### Study Design and Setting

This semirandomized controlled trial was conducted as part of the ongoing MyAgeWell project, which investigates lifestyle-based strategies to promote cognitive health among older adults in the Klang Valley, Malaysia. A total of 400 community-dwelling adults aged 60 years or older completed baseline assessments within the MyAgeWell cohort. Participants were recruited from senior centers and residential communities across the Klang Valley [17,18].

Of these, a subset was invited to participate in the present 6-month intervention trial based on willingness to engage in structured intervention sessions, availability during the intervention period, and feasibility considerations specific to the intervention modalities (eg, access to internet-enabled devices for online cognitive sessions). Individuals who did not meet these requirements or declined participation continued in the observational cohort but were not enrolled in the intervention trial.

Eligible participants were allocated to 1 of 4 groups (Zumba, video game–based cognitive stimulation, combined intervention, or control group) using a semirandomized (quasi-randomized) allocation approach. Participants were primarily assigned in a sequential cycle to maintain approximate balance in group sizes. Minor adjustments were made in 24% (23/96) of participants to accommodate the logistical feasibility of intervention delivery. No allocation concealment was implemented, and neither participants nor outcome assessors were blinded to group assignment. All intervention arms shared identical eligibility criteria, and group assignment differed only by allocation procedure rather than differential enrollment criteria.

This trial evaluated the effects of weekly physical exercise (Zumba), video game–based cognitive stimulation, and a combined intervention over 6 months, with outcomes assessed both between groups (relative to a control group) and within groups (preintervention and postintervention).

### Participants

Eligible participants were cognitively normal or without cognitive impairment, including those with well-managed medical conditions such as hypertension. All participants were required to have some mobility, defined as the ability to walk at least 3 m independently, and to be able to communicate in at least 1 of the following languages: English, Malay, Mandarin, or Tamil.

Individuals were excluded if they had a diagnosis of neurodegenerative diseases such as Alzheimer or Parkinson disease, psychiatric disorders, uncorrected hearing or vision impairments, or were currently taking psychiatric medications. Participants who were immobile, required full-time caregiver assistance, or had comprehension difficulties that could interfere with study assessments were also excluded.

### Ethical Considerations

All procedures were explained to the participants, and written informed consent was obtained from all participants before study participation in accordance with the Declaration of Helsinki. The study was approved by the Sunway University Research Ethics Committee (SUREC2020/039) and registered at ClinicalTrials.gov (NCT06376656). Participant privacy and confidentiality were protected throughout the study, and all collected data were deidentified prior to analysis. Participants were informed of the study procedures, potential risks, and their right to withdraw at any time without penalty. Participants received cash compensation of RM 50 (US $12.09) for baseline and postassessment completion, RM 30 (US $7.25) per physical intervention session, and RM 10 (US $2.42) per online cognitive intervention session.

### Sample Size Considerations

This study included all eligible participants from the ongoing MyAgeWell cohort who consented to join the 6-month intervention. No formal a priori power calculation was performed, and the sample size was determined by feasibility and participant availability within the study timeline.

### Intervention Procedures

#### Adherence Assessment and Per-Protocol Classification

No participants withdrew during the 6-month intervention, and complete MoCA data were available for all participants. Per-protocol (PP) groups were defined post hoc by applying an adherence threshold of ≥50% attendance calculated across the full 6-month intervention period for each intervention component. Participants not meeting this threshold were assigned to the control or low-adherence PP group. No participants were removed, reassigned, or reallocated during the intervention period based on adherence; all reclassifications occurred analytically at the PP stage.

#### Zumba Exercise

The Zumba intervention was delivered in 19 weekly sessions of approximately 60 minutes each at View Gallery, an activity space at Sunway University. The sessions were low impact, culturally adapted, and designed to emphasize coordination, flexibility, balance, and moderate-intensity aerobic activity. All sessions were led by certified instructors trained in working with older adults. Attendance was carefully recorded to monitor adherence.

#### Video Game–Based Cognitive Stimulation

The video game–based cognitive intervention consisted of 24 weekly online sessions delivered via Zoom, each lasting 60 minutes. Participants were asked to download and use the video games on their personal devices (Android or iOS). Two commercially available games were used: Tetris, developed by Alexey Pajitnov, targeting visuospatial reasoning and problem-solving, and Aim Lab, developed by Statespace Inc., designed to train attention, processing speed, and hand-eye coordination. Attendance and performance were monitored via Zoom screen sharing, score reporting, and session time logs. The research team did not develop or own any of the software, did not receive funding or sponsorship from the software developers, and had no other financial or personal relationships with the developers. All potential conflicts of interest are disclosed in the *Conflicts of Interest* section.

#### Combined Intervention

Participants in the combined intervention group received both physical and cognitive training components. Specifically, participants attended one 60-minute Zumba session per week delivered in person and one separate 60-minute video game–based cognitive stimulation session per week delivered online via Zoom. The 2 sessions were scheduled on different days to reduce fatigue and maximize engagement. Thus, while each intervention component was matched in duration to its corresponding single-modality arm, the combined group received a greater total weekly intervention dose (120 min/wk).

#### Control Group

Participants in the control group continued with their usual daily routines and did not participate in structured exercise or cognitive stimulation.

### Outcome Measures

Cognitive function was assessed using the MoCA before and after the intervention. The MoCA, developed and published in 2005 as a brief cognitive screening tool with high sensitivity and specificity [19], assesses global cognitive function across 7 subdomains: visuospatial or executive function, naming, attention, language, abstraction, delayed recall, and orientation [20]. Both total and subdomain scores were analyzed to determine the magnitude and domain specificity of cognitive changes. Attendance was recorded for all sessions, with adherence calculated as the percentage of sessions attended relative to the total scheduled sessions.

### Statistical Analysis

Data were analyzed using IBM SPSS Statistics (version 27). Descriptive statistics summarized participants’ demographic characteristics. Normality was assessed using the Shapiro-Wilk test, skewness, and kurtosis. Although MoCA before and MoCA after scores showed mild deviations from normality, the MoCA change score was approximately symmetric (skewness=0.13, kurtosis=0.30, Shapiro-Wilk *P*=.02), and Levene test indicated homogeneity of variances (*P*=.15). Given the sample size (n=96) and robustness of ANOVA to mild nonnormality, both parametric and nonparametric analyses were conducted.

A 2-way mixed ANOVA (time × group) was performed to examine changes in MoCA scores from baseline to postintervention across the 4 groups. Kruskal-Wallis and Wilcoxon signed rank tests were conducted as nonparametric sensitivity analyses to confirm the robustness of the findings.

Between-group comparisons of change scores were performed using Kruskal-Wallis tests, followed by Mann-Whitney *U* tests with Bonferroni correction for pairwise comparisons. Effect sizes were reported as *r*=|*Z*|/√*N* for Wilcoxon and Mann-Whitney *U* tests and as η² for Kruskal-Wallis tests, interpreted as small (0.1), medium (0.3), or large (0.5).

Associations between attendance and MoCA change were examined using Spearman rank correlation. To further explore the predictive value of attendance, multiple linear regression analyses were conducted to assess whether attendance variables significantly predicted changes in MoCA scores. In addition, receiver operating characteristic (ROC) analyses were performed to evaluate the discriminative ability of attendance percentage in identifying cognitive responders (defined as ≥2-point MoCA improvement). Statistical significance was set at *P*<.05.

## Results

### Participant Flow and Group Allocation (Intention-to-Treat and PP Analyses)

#### Participant Flow Overview

A subset of 96 participants from the MyAgeWell cohort participated in the 6-month intervention involving physical activity (Zumba), cognitive stimulation (video game–based), or combined physical and cognitive stimulation, following selection based on willingness, availability, and intervention-specific feasibility requirements.

Participants were allocated to physical activity (Zumba; n=12), cognitive stimulation (video game; n=20), combination (n=43), and control (n=21) groups (intention-to-treat [ITT] sample, N=96). There were no dropouts during the intervention period and no missing postintervention MoCA assessments. All 96 participants completed preintervention and postintervention MoCA testing and were included in the ITT analyses.

For the PP analysis, participants were reassigned based on actual adherence, using a ≥50% attendance threshold for each intervention. This threshold was chosen as a pragmatic cutoff, representing moderate engagement while retaining an adequate sample size for analysis. Adherence at the ≥50% threshold corresponded to attending at least 10 of 19 Zumba sessions, 12 of 24 cognitive sessions, or both in the combined intervention, with each session lasting 60 minutes weekly. Although higher thresholds (eg, 70%‐80%) [21] are commonly applied in medication adherence and clinical trials as a conservative definition of “good adherence,” previous studies on lifestyle and cognitive interventions have reported wide variability in attendance rates, with thresholds ranging from 50% to 80% [22,23]. Sensitivity analyses at higher thresholds in this study showed similar overall patterns but reduced analytic stability. The ≥50% attendance threshold was applied only after completion of the intervention to define analytic PP groups and did not influence intervention delivery or participant participation. Participants who did not meet the ≥50% attendance threshold, including those originally allocated to the control arm and those with partial but insufficient intervention exposure, were classified into a control or low-adherence PP group. Exposure below this level was considered unlikely to produce a meaningful intervention effect.

#### Movement of Participants From ITT Groups to PP Groups

**Figure 1.**
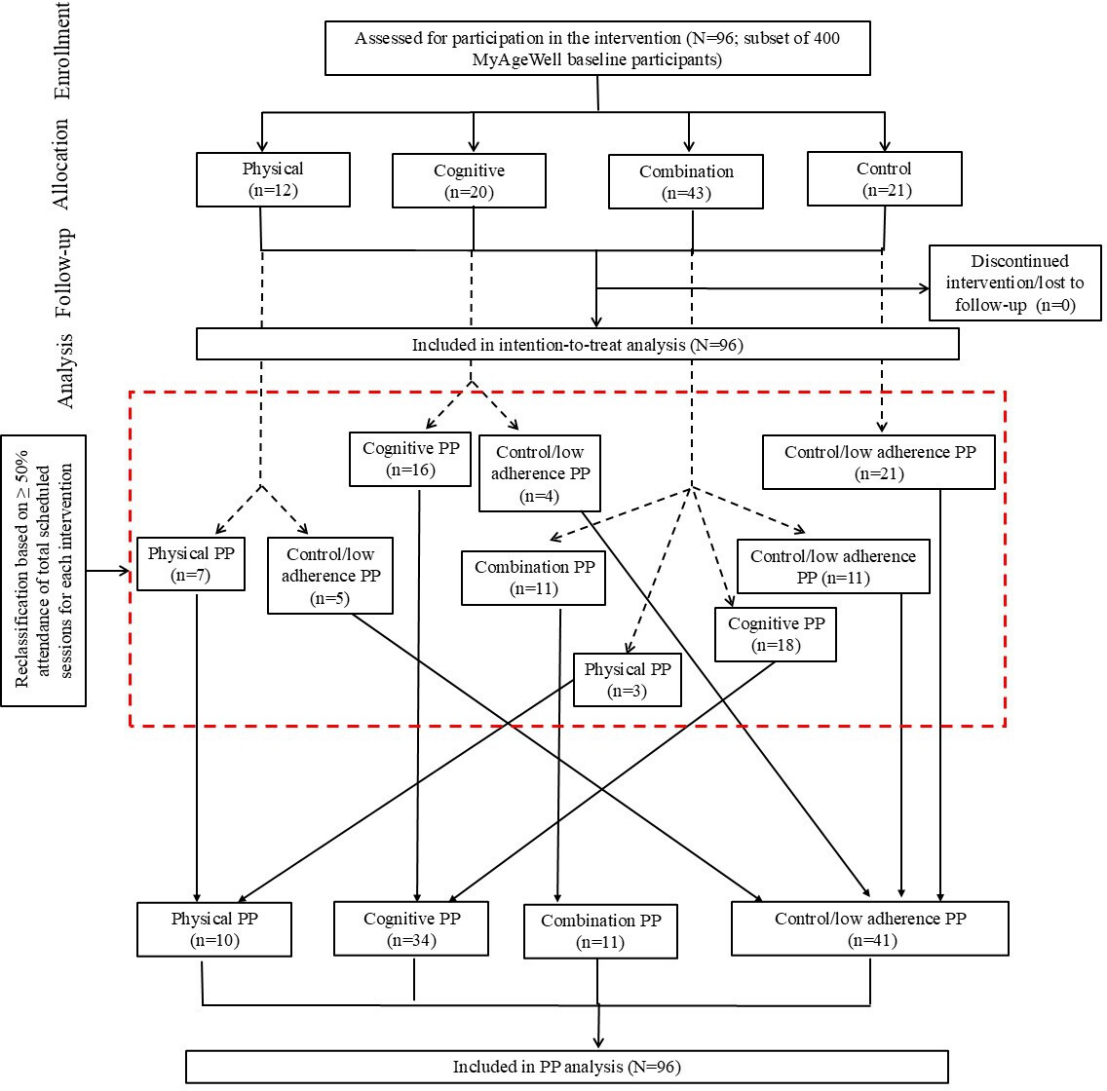
Participant flow diagram (adapted CONSORT [Consolidated Standards of Reporting Trials] for quasi-randomized trial). Of 400 participants who completed baseline assessments in the MyAgeWell cohort, a subset of 96 were enrolled in the present 6-month intervention study and allocated to physical (n=12), cognitive (n=20), combination (n=43), and control (n=21). No participants dropped out, and no postintervention Montreal Cognitive Assessment data were missing. Per-protocol reclassification (dashed red box) was based on ≥50% attendance for each intervention component and produced PP groups of physical (n=10), cognitive (n=34), combination (n=11), and control or low adherence (n=41). The 96 participants were a subset of the larger MyAgeWell cohort (N=400). PP: per-protocol.

In the ITT physical group (n=12), 7 participants attended at least 50% of the Zumba sessions and were therefore retained in the physical PP group, while the remaining 5 participants, who attended less than 50%, were reclassified into the control or low-adherence PP group. In the ITT cognitive group (n=20), 16 participants achieved at least 50% adherence to the video game sessions and remained in the cognitive PP group, whereas 4 participants with lower adherence were reassigned to the control or low-adherence PP group. In the ITT combination group (n=43), 11 participants met the adherence threshold for both Zumba and video game components and continued in the combination PP group. From the ITT combination group, 3 participants who adhered only to Zumba were reassigned to the physical PP group, 18 participants who adhered only to the video game component were moved to the cognitive PP group, and the remaining 11 participants, who did not achieve at least 50% adherence to either component, were reassigned to the control or low-adherence PP group. All 21 participants in the ITT control group remained in the control or low-adherence PP group. Following reclassification, the PP sample comprised physical (n=10), cognitive (n=34), combination (n=11), and control or low-adherence (n=41) groups. No participants were excluded from ITT analyses. This detailed reporting clarifies how participants transitioned from their original ITT allocation to the PP groups, ensuring transparency in how adherence influenced the final analytic samples. Figure 1 illustrates the participant flow diagram (adapted CONSORT [Consolidated Standards of Reporting Trials] for quasi-randomized trial; Checklist 1).

### Baseline Characteristics

Baseline characteristics were assessed according to the original group allocation. Table 1 presents participants’ demographic characteristics and baseline MoCA scores by intervention group. Participants’ median age was 69 (IQR 65-72) years and did not differ significantly across groups (Kruskal-Wallis *H*=0.277, *P*=.96). MoCA scores at baseline were similar across groups (*H*=1.131, *P*=.77), with an overall median of 26 (IQR 25-28). Besides, although female participants comprised a larger proportion of the sample (63/96, 65.6%), no statistically significant differences in sex distribution were observed across intervention groups (*χ*²_3_=1.684, *P*=.64). Education level differed significantly across groups (*χ*²_18_=55.632, *P*<.001) and represents a potential confounding factor given its known association with MoCA performance. The cognitive group had the highest proportion of participants with a bachelor’s degree (12/20, 60%), whereas the combination group included a higher proportion of participants with a master’s degree (8/43, 18.6%).

**Table 1.** Demographic characteristics and baseline Montreal Cognitive Assessment scores of participants by intervention group.^a^

Variable	Physical (n=12)	Cognitive (n=20)	Combination (n=43)	Control (n=21)	Total (N=96)	*P* value
Age (y), median (IQR)	68 (66-71)	69 (66-72)	68 (65-71)	69 (65-73)	69 (65-72)	.96^b^
Sex, n (%)						.23^c^
Female	9 (75.0)	13 (65.0)	31 (72.1)	10 (47.6)	63 (65.6)	
Male	3 (25.0)	7 (35.0)	12 (27.9)	11 (52.4)	33 (34.4)	
Education level, n						<.001^d^
No education	3	0	1	0	4	
Primary	3	0	4	1	8	
Secondary	3	1	12	9	25	
Preuniversity/diploma	0	7	7	5	19	
Bachelor’s	1	12	7	4	24	
Master’s	0	0	8	2	10	
Unknown	2	0	4	0	6	
Hypertension, n (%)	3 (25.0)	5 (25.0)	6 (14.0)	5 (23.8)	19 (19.8)	.64^d^
MoCA^e^ before, median (IQR)	26 (25-27)	26 (24-28)	26 (25-27)	26 (23-29)	26 (25-28)	.77^b^

a *P*<.05 was considered statistically significant.

b Kruskal-Wallis test for continuous variables (age and MoCA before).

c Chi-square test for sex distribution (male + female) across groups.

d Chi-square test for categorical variables (education level and hypertension) across groups.

e MoCA: Montreal Cognitive Assessment.

### Descriptive Statistics and Normality

At baseline, participants demonstrated a median MoCA score of 26 (IQR 25-28), with slight negative skew (−0.877) and moderate kurtosis (1.079). The Shapiro-Wilk test indicated significant deviation from normality (*P*<.001), but the distribution was considered suitable for further analysis. Postintervention, the median MoCA score increased to 27 (IQR 26-29), showing moderate negative skew (−1.045) and kurtosis (1.210), with Shapiro-Wilk *P*<.001. The distribution of MoCA change scores (after – before) was approximately normal (mean 0.84, SD 2.30, skewness=0.125, kurtosis=0.304, Shapiro-Wilk *P*=.02), and Levene test confirmed homogeneity of variances across intervention groups (*P*=.15). These results justified the use of both parametric (mixed ANOVA) and nonparametric (Wilcoxon signed rank, Kruskal–Wallis, and Mann-Whitney *U*) analyses.

### Mixed ANOVA (Time × Group Effects)

A 2×4 mixed ANOVA was conducted to examine the effects of time (pre vs post) and group (physical, cognitive, combination, and control) on overall MoCA scores (Table 2), with participants analyzed according to their original group allocation (ITT), irrespective of adherence level. The analysis revealed a significant main effect of time (*F*_1,92_=7.47, *P*=.008, η²=0.075), indicating that participants demonstrated a significant improvement in cognitive performance from preintervention to postintervention across all groups.

**Table 2.** Mixed ANOVA results for global Montreal Cognitive Assessment score.

Effect	Test statistic, *F* (*df*)	*P* value	Effect size (η^2^)	Interpretation
Time	7.47 (1, 92)	.008^a^	0.075	Significant main effect
Group	1.28 (3, 92)	.29	0.040	Not significant
Time × group	0.57 (3, 92)	.64	0.018	Not significant

a * P*<.05 was considered statistically significant.

**Figure 2.**
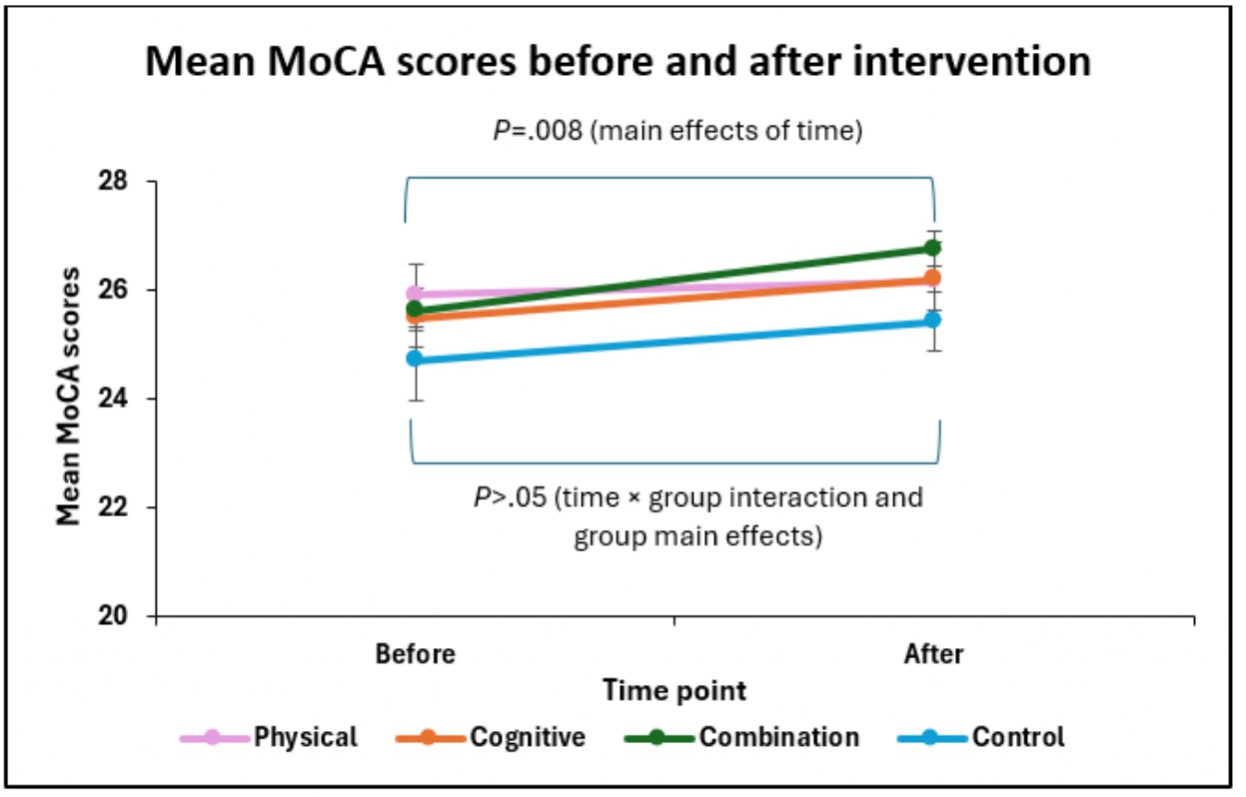
Mean MoCA scores before and after the intervention across groups. Data represent group mean (SEM). A significant main effect of time was observed (*F*_1,92_=7.47, *P*=.008, η^2^=0.075), indicating overall improvement in cognitive performance from preintervention to postintervention. The time × group interaction and group main effects were nonsignificant (*P*>.05), suggesting similar gains across all intervention groups. MoCA: Montreal Cognitive Assessment.

However, the time × group interaction was not significant (*F*_3,92_=0.57, *P*=.64, η²=0.018), suggesting that the magnitude of cognitive improvement did not differ significantly among the intervention types. Similarly, the between-group main effect was nonsignificant (*F*_3,92_=1.28, *P*=.29, η²=0.040), indicating no baseline or overall mean differences between the 4 groups. Figure 2 shows the mean MoCA scores for each group before and after the intervention, illustrating a general upward trend across time regardless of intervention condition. Although the combination group showed a numerically larger increase from preassessment to postassessment, the time × group interaction was not significant, indicating comparable improvements across groups.

### ITT Analysis: Cognitive Outcomes

#### Within-Group Changes (Wilcoxon Signed Rank Test)

Within-group comparisons of global cognitive performance were assessed using Wilcoxon signed rank tests for each intervention arm. Table 3 presents the preintervention and postintervention MoCA scores, corresponding change values, test statistics, and effect sizes.

**Table 3.** Within-group Montreal Cognitive Assessment score changes (intention-to-treat analysis, Wilcoxon signed rank test).

Group	MoCA^a^ before, median (IQR)	MoCA after, median (IQR)	Median change	*Z*	*P* value^b^	Effect size (*r*)	Interpretation
Physical (n=12)	26 (25-27)	26 (25-28)	0.00	−0.59	.56	0.17	NS^c^
Cognitive (n=20)	26 (24-28)	26 (24-28)	0.00	−0.94	.35	0.21	NS
Combination (n=43)	26 (25-27)	27 (26-28)	+1.0	−3.55	<.001	0.54	Significant
Control (n=21)	26 (23-29)	26 (25-27)	0.00	−1.22	.22	0.27	NS
Total (N=96)	26 (25-28)	27 (26-29)	+1.0	3.38	<.001	0.34	Significant (overall)

a MoCA: Montreal Cognitive Assessment.

b *P*<.05 was considered statistically significant.

c NS: nonsignificant.

Across all participants, median MoCA scores increased from 26 (IQR 25-28) at baseline to 27 (IQR 26-29) postintervention, reflecting an overall improvement of +1 points (*Z*=3.38, *P*<.001, *r*=0.34), indicating a moderate effect. Among individual groups, the combination group showed a statistically significant increase in MoCA scores from 26 (IQR 25-27) to 27 (IQR 26-28) (*Z*=−3.55, *P*<.001, *r*=0.54), representing a large effect size. In contrast, the physical (median 26, IQR 25-27 to median 26, IQR 25-28), cognitive (median 26, IQR 24-28 to median 26, IQR 24-28), and control (median 26, IQR 23-29 to median 26, IQR 25-27) groups demonstrated nonsignificant improvements, with small effect sizes ranging from *r*=0.17 to 0.27. These findings suggest that while general cognitive function improved modestly across the entire sample, the combined physical and cognitive stimulation produced the most meaningful within-group enhancement.

#### Between-Group Comparisons (Kruskal-Wallis and Mann-Whitney *U* Tests)

Between-group analyses were conducted to determine whether changes in global cognition (MoCA change scores) differed significantly across intervention groups. The Kruskal-Wallis test showed no significant difference among the 4 groups (*H*=1.20, *P*=.75, η²=0.013), indicating that overall cognitive improvement did not differ meaningfully between intervention types.

Subsequent pairwise Mann-Whitney *U* tests comparing each intervention group with the control group also yielded nonsignificant results. The physical vs control comparison (*Z*=−0.208, *P*=.84, *r*=0.036), cognitive vs control (*Z*=−0.026, *P*=.98, *r*=0.004), and combination vs control (*Z*=−0.848, *P*=.40, *r*=0.11) all demonstrated small, negligible effect sizes. Taken together, these findings suggest that while participants exhibited within-group improvements over time (Figure 2), the degree of improvement did not significantly differ across intervention conditions (Table 4). Given the small and unbalanced group sizes and the use of nonparametric tests, effect sizes (*r* and η²) were reported to indicate the magnitude and direction of effects, formal 95% CIs for these nonparametric effect sizes were not calculated due to limited methodological support and reduced interpretability in small, skewed samples.

**Table 4.** Between-group comparisons of Montreal Cognitive Assessment change scores (intention-to-treat analysis).^a^

Comparison	Test statistic	*P* value	Effect size (*r*/η²)	Interpretation
Kruskal-Wallis (4 groups)	*H*=1.20	.75	η^2^=0.013	NS^b^
Physical vs control	*Z*=−0.208	.84	*r*=0.036	NS
Cognitive vs control	*Z*=−0.026	.98	*r*=0.004	NS
Combination vs control	*Z*=−0.848	.40	*r*=0.110	NS

a Effect sizes are reported as *r* or η2. CIs were not calculated for nonparametric effect sizes due to small and uneven group sizes. *P*<.05 was considered statistically significant.

b NS: nonsignificant.

#### MoCA Subdomain Analysis (Wilcoxon Within Each Group)

To explore domain-specific cognitive effects, Wilcoxon signed rank tests were conducted for each of the 7 MoCA subdomains within the 4 intervention groups (Table 5). In the physical group, a significant improvement was observed in the language domain (*P*=.004), whereas other subdomains such as visuospatial, naming, attention, abstraction, memory, and orientation showed no significant changes.

**Table 5.** Wilcoxon signed rank test results for Montreal Cognitive Assessment subdomains by group.^a^

MoCA^b^ subdomain	Physical (*P* value)	Cognitive (*P* value)	Combination (*P* value)	Control (*P* value)	Interpretation
Visuospatial	.77	.64	.03	.81	Improved only in combination group
Naming	.32	.56	.06	>.99	NS^c^ across all groups
Attention	.56	.15	.56	.48	NS across all groups
Memory	.62	.21	.83	.55	NS across all groups
Language	.004	.05	.02	.79	Improved in physical and combination groups; trend in cognitive group
Abstraction	.32	.71	.13	.48	NS across all groups
Orientation	.38	.06	.11	.29	Trend in cognitive group only

a *P*<.05 was considered statistically significant.

b MoCA: Montreal Cognitive Assessment.

c NS: nonsignificant.

In the cognitive group, there were nonsignificant trends toward improvement in language (*P*=.05) and orientation (*P*=.06), suggesting possible subtle gains that did not reach statistical significance. Participants in the combination group demonstrated significant improvements in both visuospatial (*P*=.03) and language (*P*=.02) domains, indicating that combining physical and cognitive activities may yield broader cognitive benefits. In contrast, the control group exhibited no significant changes in any MoCA subdomain across the intervention period.

### PP Analysis: Cognitive Outcomes

#### Overall Group Comparisons

A Kruskal-Wallis test indicated no significant difference in MoCA change scores among the 4 PP groups (*H*=3.275, *df*=3, *P*=.35), with a small effect size (η²=0.034). Although not statistically significant, mean rank trends showed that participants in the cognitive and combination groups tended to exhibit slightly greater improvements compared with the physical and control or low adherence groups.

#### Within-Group Changes (Wilcoxon Signed Rank Tests)

Within-group comparisons of preintervention and postintervention MoCA scores are presented in Table 6. Significant improvements were observed in the cognitive (*Z*=−2.693, *P*=.007, *r*=0.46) and combination (*Z*=−2.200, *P*=.03, *r*=0.66) groups, reflecting moderate-to-large effect sizes. An increase of ≥2 points on the MoCA was considered a meaningful improvement. Using this criterion, participants in the cognitive and combination groups demonstrated median gains of +2 points, consistent with the definition of a cognitive responder. The physical (*Z*=−0.144, *P*=.89, *r*=0.05) and control or low adherence (*Z*=−1.468, *P*=.14, *r*=0.23) groups did not show significant changes. These findings suggest that interventions involving cognitive engagement, either independently or in combination with physical activity, led to more pronounced cognitive benefits.

**Table 6.** Within-group changes in Montreal Cognitive Assessment scores by intervention group (per-protocol analysis).

Group	MoCA^a^ before, median (IQR)	MoCA after, median (IQR)	Median change	*Z*	*P* value^b^	Effect size (*r*)	Interpretation
Physical (n=10)	26 (25‐27)	26 (25‐28)	0.0	−0.144	.89	0.05	NS^c^
Cognitive (n=34)	25 (24‐26)	27 (26‐28)	+2.0	−2.693	.007	0.46	Significant
Combination (n=11)	27 (26‐28)	29 (28‐30)	+2.0	−2.200	.03	0.66	Significant
Control or low adherence (n=41)	26 (25‐27)	26 (25‐27)	+0.0	−1.468	.14	0.23	NS

a MoCA: Montreal Cognitive Assessment.

b *P*<.05 was considered statistically significant.

c NS: nonsignificant.

#### Between-Group Pairwise Comparisons

Pairwise Mann-Whitney *U* tests comparing each intervention group with the control or low adherence group were nonsignificant (Table 7). Effect sizes were small across all comparisons (*r*<0.15), indicating minimal between-group differences in the magnitude of MoCA change.

**Table 7.** Between-group Mann-Whitney *U* comparisons of Montreal Cognitive Assessment change scores (per-protocol analysis).

Comparison	Test statistic (*Z*)	*P* value^a^	Effect size (*r*)	Interpretation
Physical vs control or low adherence	−0.527	.60	0.07	NS^b^
Cognitive vs control or low adherence	−1.291	.20	0.15	NS
Combination vs control or low adherence	−0.894	.37	0.12	NS

a *P*<.05 was considered statistically significant.

b NS: nonsignificant.

#### MoCA Subdomain Analysis (Wilcoxon Within Each Group)

Domain-specific analysis using Wilcoxon signed rank tests identified selective improvements across PP intervention groups (Table 8). The physical group demonstrated a significant improvement in the language domain (*P*=.01). The cognitive group showed significant gains in both language (*P*=.03) and orientation (*P*=.03), as well as a near-significant trend in visuospatial (*P*=.07). The combination group also showed a significant improvement in language (*P*=.02). No significant changes were observed in the control or low adherence group across all subdomains. These findings indicate that interventions incorporating cognitive elements, either alone or in combination with physical activity, particularly enhanced language and orientation abilities, which are important for executive and communicative functions among older adults.

**Table 8.** Wilcoxon signed rank results for Montreal Cognitive Assessment subdomains (per-protocol analysis).^a^

MoCA^b^ subdomain	Physical (*P* value)	Cognitive (*P* value)	Combination (*P* value)	Control/low adherence (*P* value)	Interpretation summary
Visuospatial	.59	.07	.66	.22	Trend in cognitive group
Naming	.32	.32	.32	>.99	NS^c^ across all groups
Attention	.78	.13	.66	.51	NS across all groups
Memory	.76	.81	>.99	.89	NS across all groups
Language	.01	.03	.02	.62	Improved in physical, cognitive, and combination groups
Abstraction	.66	.56	.32	.32	NS across all groups
Orientation	.41	.03	.26	.44	Improved in cognitive group

a *P*<.05 was considered statistically significant.

b MoCA: Montreal Cognitive Assessment.

c NS: nonsignificant.

### MoCA Responder Analysis

When using a ≥2-point increase as the criterion for meaningful improvement, 37.5 (36/96) participants were classified as responders, while 62.5% (n=60) participants were nonresponders. Responders were most common in the cognitive and combination groups, supporting their greater within-group gains.

### Attendance and Cognitive Change

Spearman rank correlation analyses revealed weak and nonsignificant relationships between attendance and MoCA improvement. Video game attendance showed a small positive correlation with MoCA change (*r*=0.192, *P*=.06), while total attendance was similarly weak (*r*=0.159, *P*=.12). Zumba attendance was not significantly associated with cognitive improvement.

**Figure 3.**
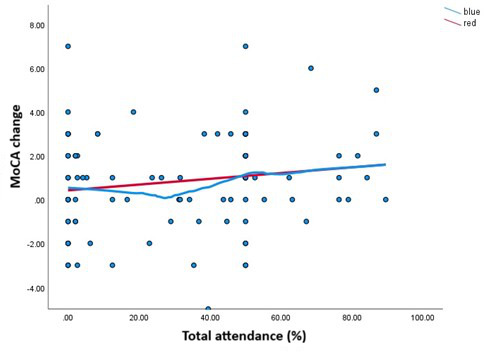
Relationship between total attendance (%) and MoCA change. Scatter plot illustrating the association between total attendance (%) and change in MoCA scores among participants. Linear (red) and locally weighted scatterplot smoothing (blue) lines are shown. Both lines indicate a weak positive trend, consistent with the nonsignificant correlation and regression analyses. MoCA: Montreal Cognitive Assessment.

Regression analysis further confirmed that attendance variables were not significant predictors of MoCA change (*P*=.18). ROC analysis indicated low discriminative accuracy of attendance measures in identifying cognitive responders, with area under the curve (AUC) values ranging from 0.45 to 0.59, suggesting poor predictive utility. Figure 3 illustrates the relationship between total attendance (%) and MoCA change. Linear (red) and locally weighted scatterplot smoothing (blue) trend lines show a weak upward trajectory, consistent with the nonsignificant statistical findings.

## Discussion

This semirandomized trial found that physical (Zumba), cognitive (video game), and combined interventions were associated with within-group improvements in global cognitive function among older adults in Klang Valley, Malaysia. The combined intervention demonstrated the largest magnitude of improvement, although between-group differences were not statistically significant. Patterns of improvement suggested that adherence may play an important role in achieving meaningful cognitive benefits.

Cognitive impairment and age-related decline substantially affect daily functioning and independence in older adults. With rapid global aging and increasing dementia prevalence, effective preventive strategies are urgently needed [15]. Pharmacological approaches show limited success and are often accompanied by adverse effects [24-26], prompting growing interest in nonpharmacological interventions targeting cognitive frailty [27-31]. Despite this progress, the comparative effectiveness of different intervention modalities and their combinations remains uncertain [9]. Our study addresses this gap by examining physical, cognitive, and combined interventions on global and domain-specific MoCA performance, providing insights into strategies to preserve cognitive health in later life.

At baseline, there were no significant differences across groups in age (*P*=.96), sex (*P*=.23), MoCA scores before intervention (*P*=.77), or hypertension prevalence (*P*=.64), indicating a well-balanced distribution among participants. Recently, our research team examined the association between C-reactive protein (CRP) levels and various demographic factors. The analysis demonstrated that CRP concentrations were significantly correlated with gender, ethnicity, and BMI (*P*<.05), but not with age or economic status [18]. CRP is an inflammatory biomarker that plays a pivotal role in the pathophysiology of age-related conditions, including cognitive decline.

However, a significant between-group difference was observed in educational attainment (*P*<.001), which is a well-established determinant of cognitive reserve and MoCA performance. The cognitive group had the highest proportion of participants with a bachelor’s degree, while the combination group included a relatively higher proportion with postgraduate education. This imbalance may have influenced both baseline cognitive performance and responsiveness to intervention, thereby complicating causal interpretation of between-group comparisons.

Although educational attainment differed across groups at baseline, the modest sample size and small subgroup numbers limited the feasibility of conducting covariate-adjusted or stratified analyses to account for potential education effects. Consequently, the observed intervention effects may have been influenced by baseline differences in cognitive reserve associated with education. Nevertheless, prior research suggests that cognitively engaging and socially meaningful interventions can benefit older adults across a broad range of educational backgrounds [32]. These considerations highlight the need to interpret our findings cautiously and the importance of larger, well-powered trials to separate intervention effects from educational influences.

Overall, the mixed ANOVA results showed a significant improvement in cognitive performance, as demonstrated in MoCA score from preintervention to postintervention (*P*=.008), indicating that engagement in structured group activities can enhance cognitive outcomes in older adults, regardless of intervention type [33]. The time × group interaction, based on the mixed ANOVA, was not significant (*P*=.64), suggesting that the magnitude of cognitive improvement was similar across intervention types. These findings were further supported by Kruskal-Wallis and Mann-Whitney analyses in both ITT and PP approaches, which showed no significant differences in MoCA change scores in between-group comparisons or in pairwise comparisons of each intervention with the control group. Consistent with Barnes et al [34], who reported that 12 weeks of physical, cognitive, or combined activities in inactive older adults did not differ from the control groups, these findings suggest that overall engagement or the total amount of activity may be more influential than the specific type of intervention. One possible explanation is that nonspecific factors, such as social interaction, may contribute to the cognitive benefits observed [33].

The absence of significant between-group differences warrants further consideration. Several methodological and contextual factors may account for this pattern. First, the relatively small and unequal group sizes in the PP analysis, particularly the physical group (n=10), reduced statistical power to detect modest effects. Second, the improvements observed in the control group might be due to practice effects from taking the MoCA test multiple times or from nonspecific benefits such as social interaction, as previously noted by Lam et al [33], as well as incidental contact with the research team during assessments. Third, the sample’s relatively high baseline MoCA scores (mean≈25) suggest a potential ceiling effect, limiting the magnitude of observable gains among cognitively healthy participants. Finally, interindividual variability in engagement and adherence may have contributed to heterogeneous treatment responses, attenuating between-group contrasts. These explanations are consistent with prior intervention studies showing that cognitive enrichment benefits can occur across multiple activity types, but group-level differences often emerge only with larger samples or higher training intensity [35-37].

Notably, the combination group showed numerically larger within-group improvements and more consistent effect sizes across analytic approaches, while the cognitive group also demonstrated significant gains in the PP analysis. Importantly, these significant improvements were observed only among participants who achieved at least moderate adherence (≥50% attendance), indicating that sufficient intervention exposure was necessary to elicit measurable gains in global cognition. However, between-group comparisons did not reveal statistically significant differences, indicating that cognitive improvements over time were broadly comparable across intervention conditions. Within this context, the relatively larger effect sizes observed in the combined intervention, reflected by large within-group effects in both the ITT (*r*=0.54) and PP (*r*=0.66) analyses, suggest a potential advantage of multimodal engagement when adherence is maintained. These patterns are consistent with previous reports indicating that combined physical and cognitive interventions may be associated with greater improvements in global cognition compared with single-modality approaches, as observed by Yu et al [35] and Han et al [36]. Mechanistically, dual-task or multitask training that integrates physical and cognitive elements has been proposed to support cognitive function through neurophysiological pathways, including reduced bilateral prefrontal cortical oxygenation [36], increased hippocampal volume [38], and improved white matter integrity [39].

The video game-based cognitive intervention demonstrated meaningful benefits in this study, particularly among participants who maintained at least moderate adherence in the PP analysis. This aligns with the meta-analysis by Lampit et al [14], which reported that computerized cognitive training produces small but significant improvements in cognitive performance among healthy older adults. Participants engaging more consistently with the cognitive intervention showed the largest gains, highlighting the importance of adherence in achieving measurable benefits. In contrast, the physical activity group showed minimal cognitive change across both analytic approaches, suggesting that a once-weekly Zumba session may not have provided a sufficient cumulative training dose to elicit substantial cognitive improvements.

Besides, Zhang et al [40] examined the effects of all FITT-VP (frequency, intensity, time [duration], type, volume, and progression) exercise variables on cognition across healthy populations, including older adults, and found that variations in FITT-VP influence cognitive benefits. Older adults showed the greatest improvements in global cognition, executive function, and memory. Yuan et al [31] similarly reported that exercise improved cognition in older adults with cognitive frailty, particularly when conducted 3 or more times per week, with each session lasting at least 45 minutes over 12 weeks or longer. Chia et al [17] also observed that participants with higher physical activity levels exhibited significantly greater median MoCA scores compared with less active participants. Collectively, these findings emphasize that exercise characteristics, including frequency, intensity, and session duration (time), are crucial determinants of cognitive outcomes.

The present findings are consistent with recommendations from the American College of Sports Medicine and prior meta-analyses. Northey et al [41] reported that moderate-intensity exercise lasting at least 45 minutes per session on most days of the week enhances global cognition in adults aged 50 years and older. Xue et al [42] similarly found that moderate-duration, moderate-intensity exercise is associated with significant cognitive improvements among older adults. Although these studies do not explicitly recommend the FITT-VP framework, their results indirectly support its core principles, particularly frequency, intensity, and time, as key determinants of cognitive benefit.

In this study, Zumba sessions were delivered once weekly for 60 minutes over approximately 19 weeks. While session intensity was appropriate, the low frequency likely limited cumulative training dose, providing a plausible explanation for the minimal cognitive gains observed. Applying FITT-VP principles as an interpretive framework highlights the importance of optimizing exercise frequency, duration, and intensity in future interventions to ensure sufficient training stimulus for measurable cognitive improvements among older adults. While each intervention component was matched in session duration, the combined arm received a higher total weekly exposure, and this difference in intervention “dose” was considered when interpreting between-group comparisons.

The study was further extended with subdomain analyses under both analytic approaches (ITT and PP) to explore domain-specific responsiveness to the different interventions. The results showed that the language domain, which included assessments of verbal, categorical, and phonological fluency [43], demonstrated the most consistent improvement across all intervention groups. This suggests that verbal fluency and communication-related cognitive processes are particularly sensitive to structured and socially interactive activities. These findings are also supported by previous studies, although the results varied depending on the intervention type. Nocera et al [44] reported that aerobic exercise improved verbal fluency, whereas cognitive training was associated with decreased or nonsignificant improvement in this domain. In contrast, Calatayud et al [45] demonstrated that a comprehensive cognitive stimulation program significantly enhanced language proficiency, verbal fluency, and overall cognitive function. These contrasting findings indicate that the type and structure of the intervention may differentially influence specific cognitive domains.

Significant gains in the visuospatial domain were observed in the combination group in the ITT analysis, while improvements in orientation emerged in the cognitive stimulation group and reached significance in the PP analysis. These patterns suggest that distinct cognitive mechanisms may underlie the effects of different stimulation modalities. Interventions that combine physical and cognitive elements may enhance visuospatial and integrative processing, whereas purely cognitive activities may strengthen orientation. Collectively, these subdomain-level findings highlight the importance of examining domain-specific outcomes beyond total MoCA scores, as they may reveal early indicators of intervention efficacy [15].

Exploratory analyses examining adherence showed that attendance rates were not significantly correlated with MoCA improvement. While weak positive associations were observed for video game and total attendance, regression and ROC analyses showed limited predictive value for cognitive improvement. This suggests that mere attendance frequency may not fully capture the quality or engagement level of participation, underscoring the complexity of dose-response relationships in behavioral interventions. Nevertheless, adherence remains clinically relevant, as participants with consistent engagement in cognitive or combined activities demonstrated the most robust improvements in the PP analyses.

From a clinical perspective, these findings highlight the feasibility of combined physical and cognitive interventions as a practical approach for promoting cognitive engagement among older adults. While between-group differences were not statistically significant, the consistent within-group improvements and favorable subdomain patterns observed in participants with higher adherence suggest that sustained participation in structured multimodal activities may be beneficial in real-world community settings.

In relation to previous literature, the present findings are consistent with meta-analyses reporting small-to-moderate improvements in global cognition following cognitive and combined interventions [15,46]. This study adds novel insights by directly comparing physical activity, cognitive stimulation, and combined training within a single cohort, employing both ITT and PP analytic frameworks and mapping MoCA subdomains to identify domain-specific responsiveness. Such findings contribute to growing evidence that multimodal engagement may support cognitive health in aging populations, although definitive conclusions regarding superiority over single-domain training require larger, adequately powered trials.

Several limitations should be acknowledged. The semirandomized allocation and lack of blinding may have introduced expectancy effects, although baseline characteristics were comparable across groups and standardized outcome assessments were used to mitigate potential bias. Additionally, the sample size was modest and uneven across groups, limiting statistical power and precision. The intervention frequency (once weekly) and duration may have been insufficient to elicit large cognitive changes. Cognitive outcomes were based solely on the MoCA, without complementary neuropsychological or neuroimaging measures. Moreover, the study lacked long-term follow-up, preventing evaluation of the persistence of cognitive gains. Furthermore, as participants were community-dwelling older adults from a single urban region, generalizability to rural or clinical populations is limited.

Besides, adherence thresholds for the PP analysis were pragmatically defined at ≥50% attendance. Sensitivity analyses using higher cutoffs (70%‐80%) yielded directionally similar results, but small subgroup sizes reduced statistical stability, highlighting the trade-off between stricter adherence definitions and analytic feasibility in nonpharmacological interventions. Pooling participants with no intervention exposure and those with partial exposure below the ≥50% adherence threshold increased heterogeneity and may have attenuated detectable between-group differences.

Finally, formal 95% CIs for nonparametric between-group effect sizes were not calculated due to methodological constraints with small, skewed samples. Consequently, effect sizes (*r* and η²) were used to indicate magnitude and direction rather than statistical precision, and between-group findings should be interpreted cautiously.

In summary, both cognitive and combined interventions produced meaningful improvements in cognitive performance among older adults, with the strongest effects observed when adherence was maintained. Although between-group differences were not statistically significant, the consistent within-group trends and domain-specific patterns suggest practical cognitive benefits of engaging in regular, structured multimodal activities. Future research with larger samples, longer intervention durations, and multidomain cognitive assessments is warranted to confirm these findings and further clarify the mechanisms underlying differential cognitive responsiveness.

This study demonstrates that participation in video game–based cognitive stimulation, physical activity, and combined interventions was associated with within-group improvements in global cognitive performance among community-dwelling older adults. PP analyses indicated that participants who attended at least 50% of the cognitive stimulation sessions showed moderate improvements, while those who adhered to at least 50% of both the physical and cognitive components in the combined intervention demonstrated numerically larger and more consistent gains. Domain-specific analyses suggested that language, visuospatial, and orientation abilities may respond differently to intervention type. Although between-group differences were not statistically significant, consistent within-group trends support the practical value of structured engagement in cognitive and combined activities. Future studies with longer durations, higher frequency or intensity of physical activity, larger sample sizes, and multidomain cognitive assessments are needed to clarify the mechanisms underlying intervention-specific cognitive responsiveness.

## Supplementary material

10.2196/88479 Checklist 1 CONSORT-eHEALTH checklist (V 1.6.1).

## Abbreviations

CONSORT: Consolidated Standards of Reporting Trials
FIIT-VP: frequency, intensity, duration time, type, volume, and progression
ITT: intention-to-treat
MCI: mild cognitive impairment
MoCA: Montreal Cognitive Assessment
PP: per-protocol
ROC: receiver operating characteristic
